# Exploring microbial players for metagenomic profiling of carbon cycling bacteria in sundarban mangrove soils

**DOI:** 10.1038/s41598-025-89418-x

**Published:** 2025-02-08

**Authors:** Basanta Kumar Das, Ayushman Gadnayak, Hirak Jyoti Chakraborty, Smruti Priyambada Pradhan, Subhashree Subhasmita Raut, Sanjoy Kumar Das

**Affiliations:** ICAR-Central Inland Fisheries Research Institute, Barrackpore, Kolkata, 700120 India

**Keywords:** Metagenomics, Microbial, Carbon, Mangrove soils, Functional genes, Computational biology and bioinformatics, Microbiology

## Abstract

The Sundarbans, the world’s largest tidal mangrove forest, acts as a crucial ecosystem for production, conservation, and the cycling of carbon and nitrogen. The study explored the hypothesis that microbial communities in mangrove ecosystems exhibit unique taxonomic and functional traits that play a vital part in carbon cycling and ecosystem resilience. Using metagenomic analysis to evaluate microbial communities in mangrove and non-mangrove environment, evaluating their composition, functional functions, and ecological relevance. The analysis revealed distinct microbial profiles, in mangrove and non-mangrove environments, with bacteria, proteobacteria, and viruses being the most prevalent groups, with varying abundances in each environment. Functional and taxonomical analysis identified genes involved in carbon regulation, including *Triacylglycerol lipase*, *NarG*, *DsrB*, *DNA-binding transcriptional dual regulator CRP*, *Vanillate O-demethylase oxygenase*, *succinate-CoA ligase*, *Tetrahydrofolate ligase*, *Carboxylase*, *Ribulose-1*,*5-bisphosphate carboxylase/oxygenase*, *Glycine hydroxymethyltransferase*, *MAG: urease*, *Endosymbiont of Oligobrachia haakonmosbiensis*, *Ribulose bisphosphate carboxylase*, *Aconitate hydratase AcnA*, and *nitrous oxide reductase*, suggesting the metabolic versatility of these microbial communities for carbon cycling. The findings emphasize the key role of microbial activity in preserving mangrove ecosystem health and resilience, highlighting the intricate interplay between microbial diversity, functional capabilities, and environmental factors.

**Supplementary Information:**

The online version contains supplementary material available at 10.1038/s41598-025-89418-x.

## Introduction

The Sundarbans, a large contiguous mangrove ecosystem in the Bay of Bengal delta, is known for its rich biodiversity and unique ecological functions^1^. The mangrove forest acts as an insulating layer between the land and the water, which has a significant influence on the dynamics of climate change and environmental sustainability^2^. Understanding microbial carbon cycle in both environments is essential for sustainable conservation measures. Mangroves, functioning as carbon sinks, mitigate human CO_2_ emissions, while knowledge from non-mangrove ecosystems offers strategies for minimizing carbon losses in agricultural and terrestrial environments^3,4^. However, research on the intricate interactions between the microorganisms in the mangrove soils of the Sundarbans and those in nearby non-mangrove soils, particularly regarding carbon management, has been insufficient. Mangrove soils possess elevated organic matter levels, anaerobic environments, and salinity, fostering distinct microbial community’s adept in carbon sequestration and methane cycling. Conversely, non-mangrove soils, such agricultural fields or uplands, exhibit more aerated conditions and are affected by human activity, resulting in unique microbial processes^5^. According to a recent study, carbon dioxide (CO_2_) stands out among greenhouse gases as the leading cause of climate change and global warming^6^. CO_2_is predominantly linked to anthropogenic activities like burning fossil fuels and deforestation^7^. Microbial communities, especially bacteria, play an increasingly important role in moderating carbon fluxes across terrestrial ecosystems owing to their broad metabolic capacities and extensive ecological implications^8^.

Using high-throughput sequencing and analysis of microbial DNA extracted directly from environmental samples, a culture-independent approach known as metagenomics has recently become a potent tool for understanding the taxonomic composition and functional potential of diverse microbial communities^9–12^. Metagenomic studies improve our knowledge of ecosystem processes and functions by analyzing the collective genetic material in a specific habitat. This allows us to learn more about the variety, quantity, and metabolic activities of microorganisms.

Several genome studies in the past few years have shed light on the microbial communities involved in carbon cycling in marshes, peatlands, and farming soils, among other places on land^13,14^. However, few comprehensive metagenomic investigations presently concentrate on the Sundarbans mangrove ecosystem and surrounding non-mangrove soils. Understanding the microbial processes that govern carbon sequestration in these disparate environments is crucial for forecasting their reactions to environmental alterations and guiding appropriate conservation and management approaches^15^. Mangrove soils are specialized ecosystems distinguished by significant organic carbon buildup, anaerobic conditions, and tidal fluctuations, which support distinct microbial communities essential for carbon storage and methane cycle. Conversely, non-mangrove soils, including agricultural or upland regions, exhibit more aerated conditions, varied organic inputs, and anthropogenic disturbances, leading to unique microbial activities associated with carbon cycling^16^. By comparing soils from mangrove and non-mangrove areas in the Sundarbans, this research hopes to fill this informational vacuum about carbon-regulating bacteria. By analyzing the taxonomic mix, functional potential, and ecological functions of microbial communities in these ecosystems, we aim to understand the fundamental processes that influence carbon dynamics and their impact on climate control.

**Fig. 1:**
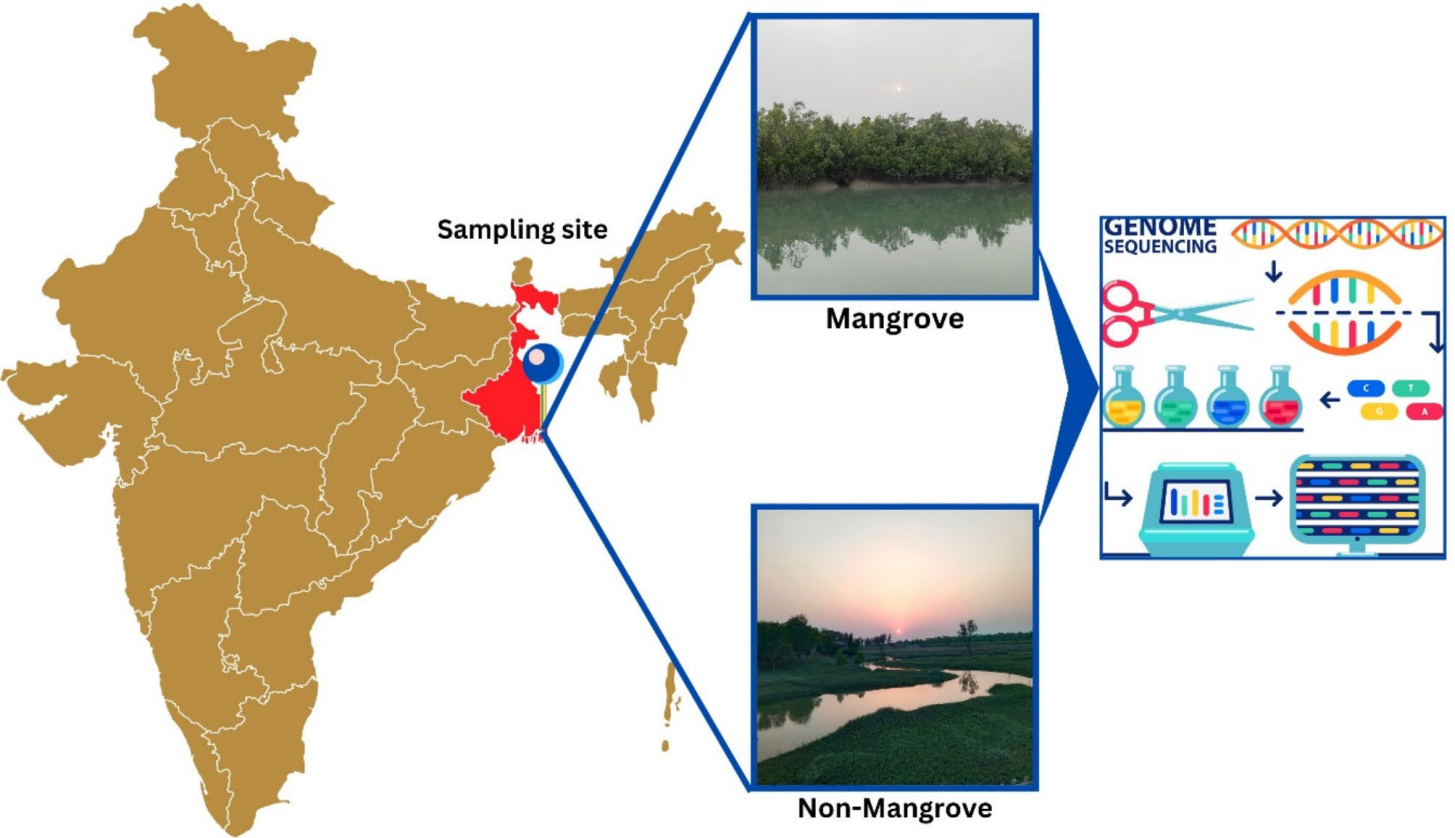
Highlights the west bengal’s sundarbans (red on map). Mangrove and non-mangrove soils are sampled. The right panel shows microbial DNA extraction, genome sequencing, and bioinformatics analysis to examine carbon cycling by microbes.

The development of high-throughput sequencing technology and bioinformatics tools for analyzing metagenomic data has allowed for a detailed investigation of microbial communities at an unprecedented level of detail^17,18^. We used these tools and methods, along with shotgun metagenomic sequencing, to investigate the genetic diversity and metabolic pathways associated with carbon metabolism in mangrove and non-mangrove soils in the Sundarbans, depicted in Fig. 1. Researchers have already found that microbes play a significant role in determining the biogeochemical cycles of carbon in mangrove environments^19,20^. The Sundarbans, as the world’s largest contiguous mangrove ecosystem and a UNESCO World Heritage Site, represents a globally significant carbon sink^21^. The unique combination of tidal flooding, saline soils, and rich biodiversity creates an exceptional natural laboratory for examining microbial roles in carbon cycle^22^. However, carbon fluxes in Sundarban mangrove and non-mangrove soils have yet to be well known, and the relative contributions of various microbial species are even less clear. This knowledge gap is vital for global carbon management and for predicting ecosystem resilience of climate change.

In addition, there is still a need for more excellent studies into how salinity, tidal flooding, and plant-microbe interactions affect the composition and function of microbial communities in mangrove ecosystems^21,23^. Recent research has shown that mangrove-associated microorganisms can adapt to changing environmental circumstances, emphasizing their resistance to environmental stresses and potential significance in ecosystem resilience and climate change adaptation^22,24^.

## Results

### Sequencing, quality-control and gene prediction

After eliminating the adaptor and low-quality sequences from the original data, we got 40,877,196 (2 × 150 bp) high-quality reads for the mangrove samples and 34,413,814 (2 × 150 bp) high-quality reads for the non-mangrove samples. The reads were further organized into scaffolds, resulting in 1,585,765 and 1,517,859 scaffolds with average sizes of 476 bp and 438 bp for the mangrove and non-mangrove samples, respectively. Gene prediction revealed the presence a total of 261,910 genes in the mangrove metagenome and 223,837 genes in the non-mangrove metagenome. It is important to note that these numbers were obtained by considering only genes with a minimum length of 500 base pairs. The quality control data is shown in Table S1, while the statistics for the assembly of Sundarban mangrove and non-mangrove are shown in Table S2.

### Microbial taxonomy of the mangrove and non-mangrove ecosystem

**Fig. 2:**
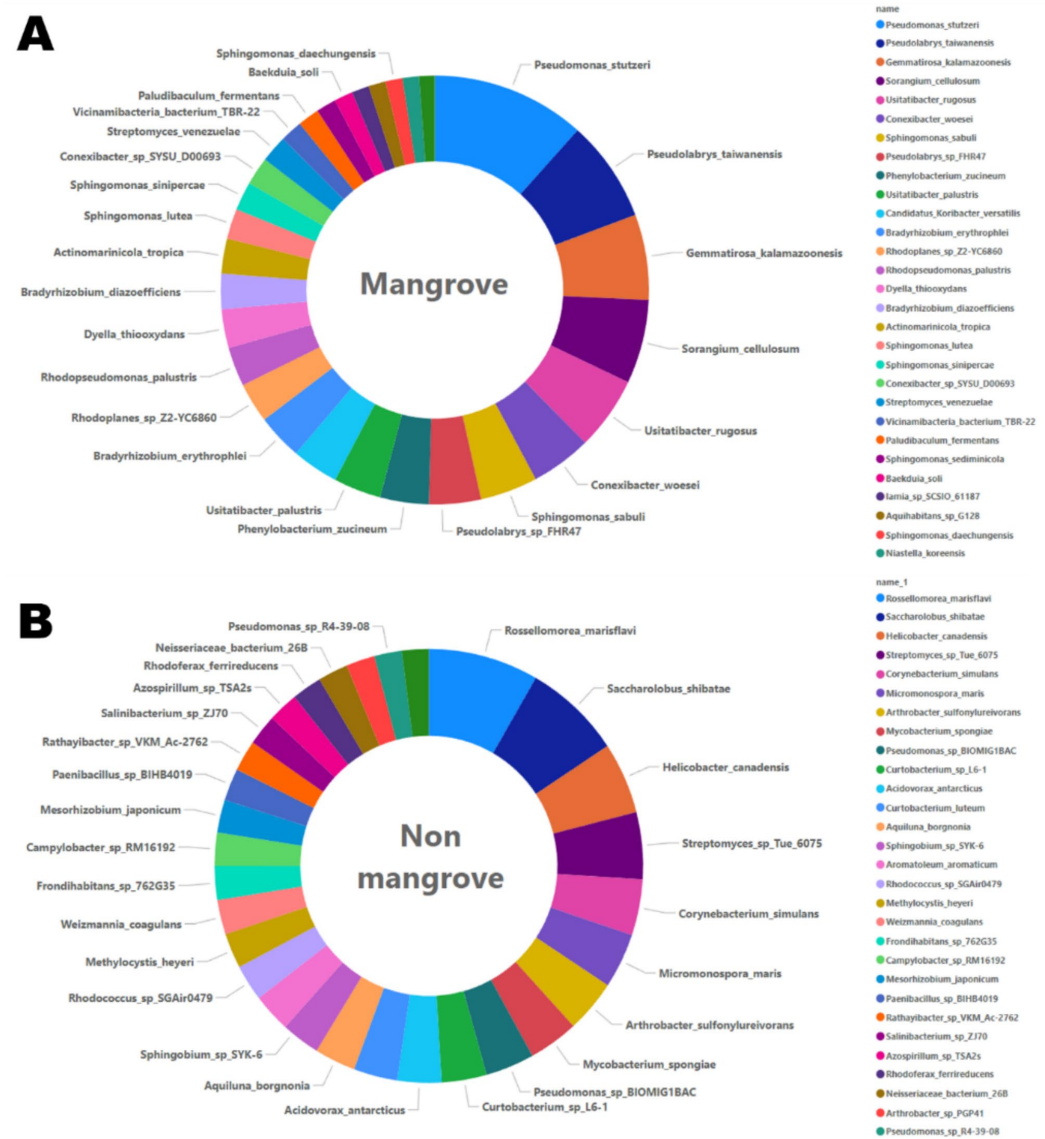
Taxonomic classification of microbial communities in (**A**) Mangrove and (**B**) Non-mangrove soils.

The taxonomic profile analysis conducted using Kraken 2 revealed that bacterial taxa constituted 39% of the microbial community in the samples of mangrove soil, whereas viruses represented just 1%. It was not possible to accurately categorize the remaining 60% of readings into a specific taxonomic group. Conversely, soil samples from non-mangrove areas showed a bacterial representation of 40% and a very little prevalence of viruses at 0.02%. Other significant microbial groups, each constituting at least 0.1% of classified reads in either environment, in this regards the top 30 taxonomical analysis ware identified for both the ecosystems based on there highest relative abundance. Specifically, in the mangrove, *Pseudomonas_stutzeri*, *Pseudolabrys_taiwanensis*, *Gemmatirosa_kalamazoonesisare*, *Sorangium_cellulosum*, and *Usitatibacter_rugosus* were the most abundant taxa, as visualized in Fig. 2A. In the non-mangrove ecosystem, the top 5 taxa included *Rossellomorea_marisflavi*,* Saccharolobus_shibatae*,* Helicobacter_canadensis*,* Streptomyces_sp_Tue_6075*,* Corynebacterium_simulans* as depicted in Fig. 2B.

### Taxonomic assignment, abundance and diversity analysis

The research focused on categorizing sequenced DNA fragments (reads or contigs) into several taxonomic groupings, including phylum, class, order, family, genus, and species. Through the comparison of these sequences with reference databases that include well-known microbial genomes, we successfully determined the identities of the species and measured the abundance of the various microbial communities found in both mangrove and non-mangrove soil samples. The organisms mentioned are *Paenarthrobacter aurescens*,* Roseobacter denitrificans*,* Pinus ponderosa*,* Komagataeibacter saccharivorans*,* Candidatus Manganitrophus*, *uncultured bacterium*, *endosymbiont*, *Halothiobacillus neapolitanus*,* Sphingomonas cynarae*, *Komagataeibacter saccharivorans*, *Bacillus subtilis*, *Desulforapulum autotrophicum*, *Escherichia coli*, *Pseudomonas sp.*, and *Roseihalotalea indica*. By following this procedure, we successfully generated a thorough taxonomic profile of the microbial communities that live in these different soil conditions. This allowed us to get useful knowledge about their composition and the possible ecological functions they may have.

The mangrove and non-mangrove soils that were examined indicate that there are different compositions of microbial communities. Actinobacteria and Firmicutes are often associated with higher GC content, while Proteobacteria and Bacteroidetes tend to have lower GC content. However, it is important to note that unique environmental factors might affect these patterns. This study, particularly highlighting the GC content differences 51.54% in mangrove and 63.96% in non-mangrove soils, and the detailed raw metrics, such as total reads, Q20/Q30 values, and duplicate rates, etc. which is depicted in Table S1. These results emphasize the complex relationship between the variety of microorganisms, their functional capacities, and the environmental conditions in both mangrove and non-mangrove soil ecosystems. They provide a fundamental comprehension for further inquiries into the ecological functions of these microorganisms and their impacts on soil health and the ability of an ecosystem to withstand and recover from disturbances.

### Carbon regulating genes identification and analysis

**Fig. 3:**
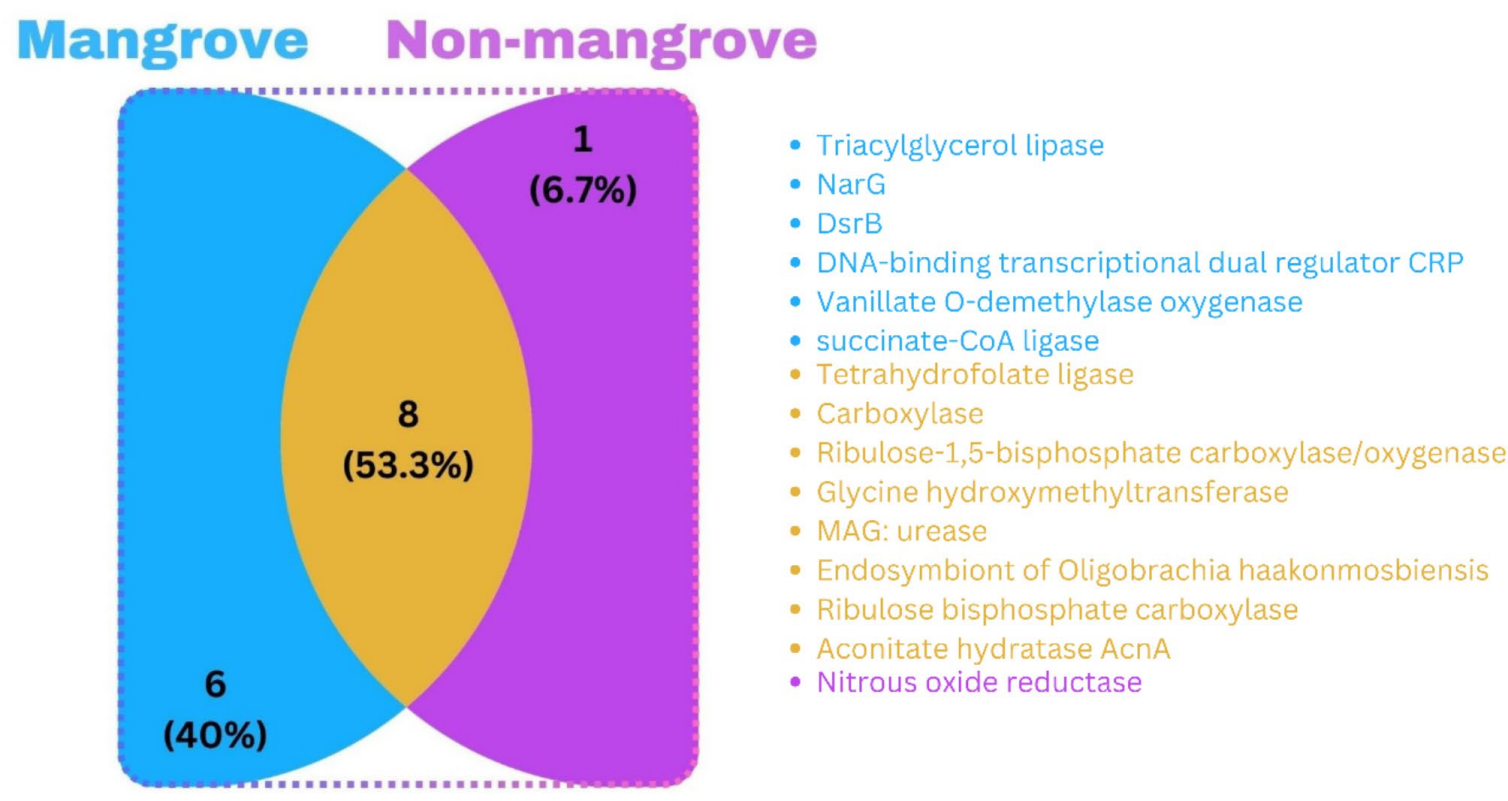
The identified CRGs, eight genes were common to both mangrove and non-mangrove soils, while six genes were unique to mangrove soils and one gene to non-mangrove soil.

A total of 15 Carbon Regulating Genes (CRGs) have been identified, with 8 genes (*Tetrahydrofolate ligase*, *Carboxylase*, Ribulose-1,5-bisphosphate carboxylase/oxygenase, *Glycine hydroxymethyltransferase*, *MAG: urease*, *Endosymbiont of Oligobrachia haakonmosbiensis*, *Ribulose bisphosphate carboxylase*, and *Aconitate hydratase AcnA*) being common in all sites. Additionally, 6 genes (*Triacylglycerol lipase*, *NarG*, *DsrB*, *DNA-binding transcriptional dual regulator CRP*, *Vanillate O-demethylase oxygenase*, and *succinate-CoA ligase*) are uniquely identified in mangrove soil, while 1 gene (*nitrous oxide reductase*) is unique to non-mangrove soil. These findings are depicted in Fig. 3 Venn diagram.

**Fig. 4:**
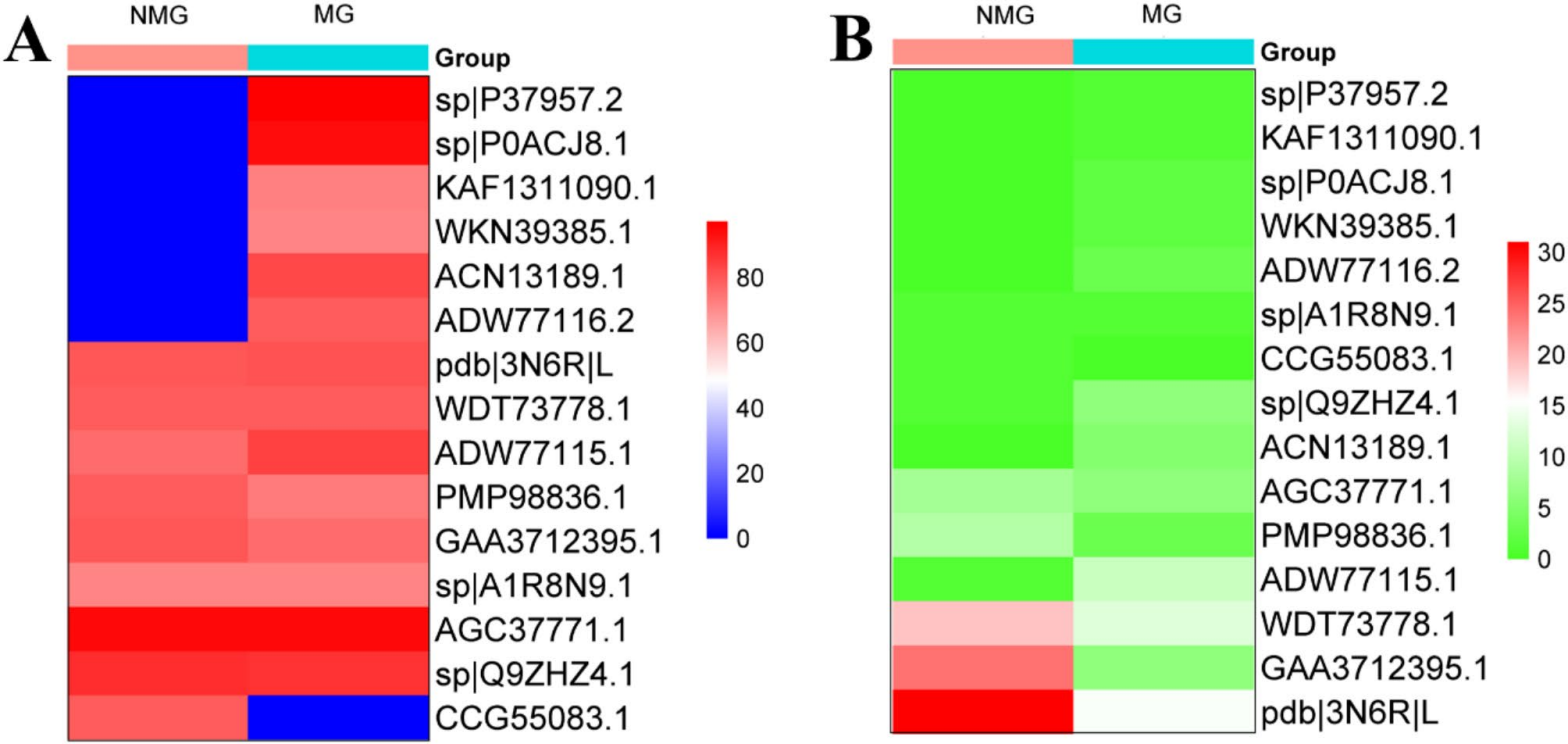
The Heatmap representation of top 15 carbon regulating genes where (**A**) represents Percent identity and (**B**) represents frequency of observation of carbon regulating genes.

The CRG analysis provided information on the percentage of similarity and the frequency of observation of both mangrove and non-mangrove species, as shown in Fig. 4A & B heatmaps. The heatmaps depict the CRGs found in the samples, providing insightful data on the functional potential and ecological functions of the microbial communities in the investigated environment. The CRGs provide a comprehensive investigation of the microbial community’s participation in carbon cycling in the investigated ecosystem. Table 1 displays the annotation of carbon-regulating microorganisms and plants.

**Fig. 5:**
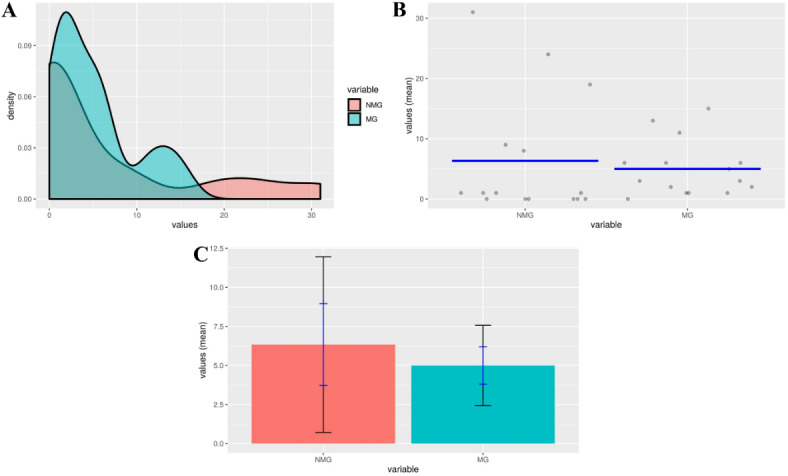
The data on carbon regulating genes (**A**) Density plots depict the distribution and overlap of gene expression values in Sundarban MG and NMG groups. (**B**) Scatter plot provides an initial overview of the data distribution. (**C**) Comparison of median, quartiles, and potential outliers between the two groups displays in Boxplot.

The NMG group had a mean of 6.333, a standard deviation of 10.161, a standard error of 2.623, and a margin of error of 5.627. The MG group had a mean of 5.000, a standard deviation of 4.645, a standard error of 1.199, and a margin of error of 2.572. The p-value, which is higher than the commonly used significance threshold of 0.05 shown in supplementary Table S3, indicate that the differences in CRGs between the Sundarban mangrove and non-mangrove groups are statistically significant at a confidence level of 95%. Similarly, the t-test shown in Fig. 5A demonstrates the density distribution of carbon regulating genes in both the Sundarban MG and NMG groups, revealing the extent of overlap and dispersion of results. Figure 5B displays the first plot that offers an initial picture of the data distribution prior to doing in-depth statistical analysis. Figure 5C presents a boxplot that compares the carbon regulating genes between the two groups. It emphasizes the median values, quartiles, and any possible outliers.

**Table 1:** CRGs along with their corresponding bacteria or plant origin, annotated enzymes, and associated organisms, with accession numbers.

Accession no.	Virus/bacteria/enzyme	Name	Organism
k141_793650-sp|A1R8N9.1	Bacteria	*Tetrahydrofolate ligase*	Paenarthrobacter aurescens TC1
k141_512908-pdb|3N6R|L	Bacteria	*Carboxylase*	Roseobacter denitrificans OCh 114
k141_145717-AGC37771.1	Plant	*Ribulose-1*,*5-bisphosphate carboxylase/oxygenase*	Pinus ponderosa var. scopulorum
k141_11437-PMP98836.1	Bacteria	*Glycine hydroxymethyltransferase*	Komagataeibacter saccharivorans
k141_75295-WDT73778.1	Bacteria	*MAG: urease*	Candidatus Manganitrophus sp.
k141_318279-CCG55083.1	Bacteria	*Nitrous oxide reductase*	uncultured bacterium
k141_999151-ADW77115.1	Bacteria	*Endosymbiont of Oligobrachia haakonmosbiensis*	endosymbiont of Oligobrachia haakonmosbiensis
k141_716870-sp|Q9ZHZ4.1	Bacteria	*Ribulose bisphosphate carboxylase*	Halothiobacillus neapolitanus c2
k141_979967-GAA3712395.1	Bacteria	*Aconitate hydratase AcnA*	Sphingomonas cynarae
PMP98836.1	Bacteria	*Glycine hydroxymethyltransferase*	Komagataeibacter saccharivorans
sp|P37957.2	Bacteria	*Triacylglycerol lipase*	Bacillus subtilis subsp. subtilis str. 168
ACN13189.1	Bacteria	*NarG*	Desulforapulum autotrophicum HRM2
ADW77116.2	Bacteria	*DsrB*	endosymbiont of *Oligobrachia haakonmosbiensis*
sp|P0ACJ8.1	Bacteria	*DNA-binding transcriptional dual regulator CRP*	*Escherichia coli* K-12
KAF1311090.1	Bacteria	*Vanillate O-demethylase oxygenase*	Pseudomonas sp. SG-MS2
WKN39385.1	Bacteria	*Succinate–CoA ligase*	*Roseihalotalea indica*

### Analysis of functional annotation

**Fig. 6:**
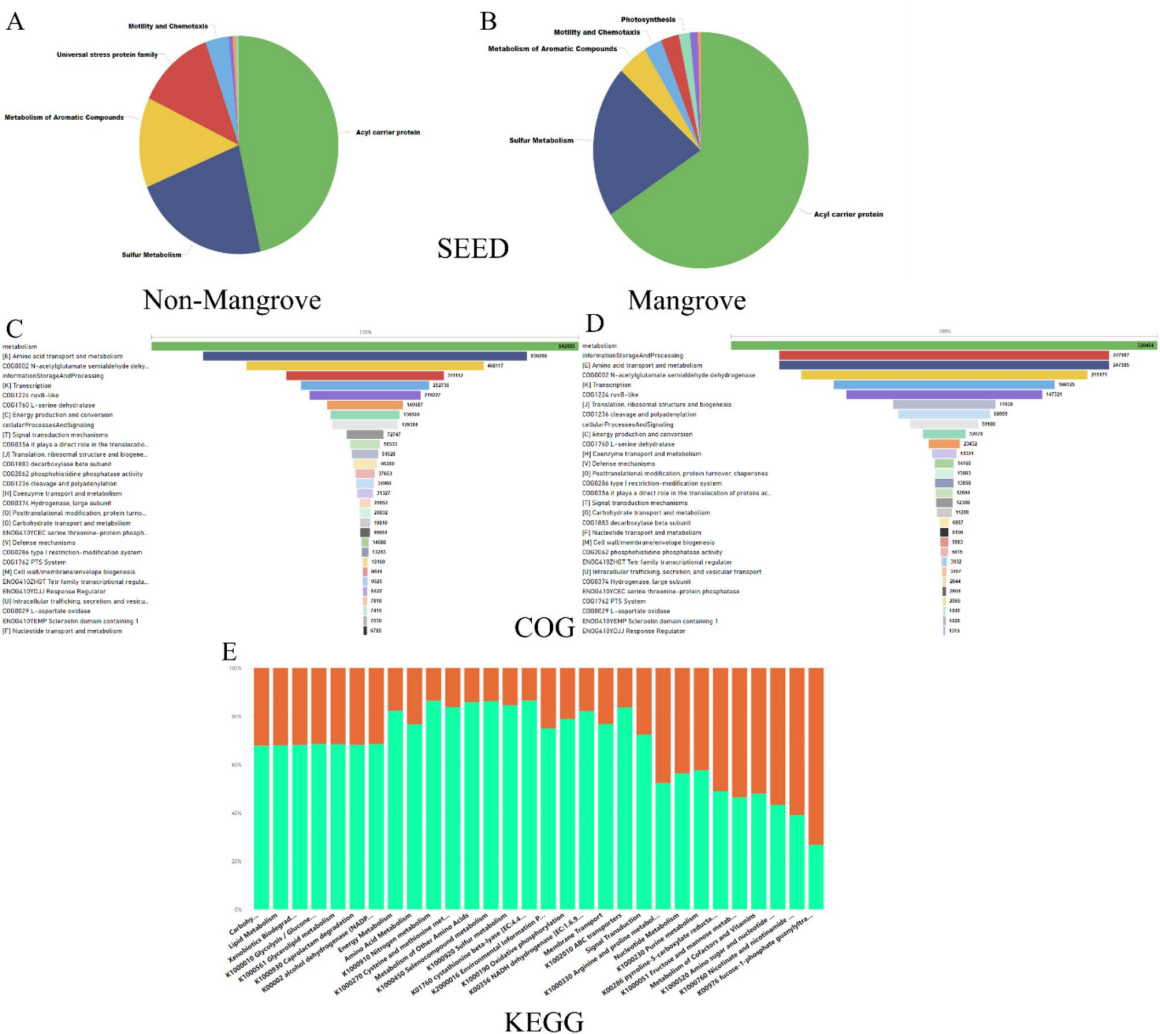
Differential functional expression analysis in various soil conditions Non-mangrove and Mangrove displayed unique patterns in SEED (**A** and **B**), COG (**C** and **D**), and KEGG (**E**). Light green indicates non-mangrove, whereas orange indicates mangrove.

The analysis of seed annotation data has shown a significant variation in the level of Acyl Carrier Protein (ACP) across various soil conditions. Acyl Carrier Protein is particularly prevalent in the soils linked to mangrove habitats, suggesting a possibly crucial function in the metabolic processes tailored to these distinct environments. Conversely, non-mangrove soils exhibit much lower amounts of Acyl Carrier Protein, indicating a decreased need or the presence of alternate routes in non-mangrove environments, shown in Fig. 6A & B. The differences in the microbial gene abundance highlights the adaptive biochemical pathways used by species in mangrove environments to flourish under certain conditions. COG analysis in this context has shown significant differences in functional gene categories across soils in mangrove and non-mangrove areas. Genes associated with energy production and conversion are significantly abundant in mangrove habitats, indicating the need to maintain energy equilibrium in this demanding ecosystem. On the other hand, soils that are not mangroves have a higher number of metabolism related to preserving and analyzing information, as seen in Fig. 6C & D. This indicates that these habitats have different functional priorities. The observed gene distribution highlights potential functional capacities of microbial communities in mangrove and non-mangrove soils. The analysis of KEGG pathway data has shown significant variations in the metabolic pathways across different soil conditions. The pathways associated with metabolism and biosynthesis are notably increased in mangrove soils, indicating a heightened involvement of these processes in adapting to the distinctive challenges of the mangrove environment. In contrast, non-mangrove soils show reduced abundance levels of these pathways, as seen in Fig. 6E, indicating a distinct metabolic emphasis or alternate processes in these settings. The differences in the pathway reveals the distinct metabolic mechanisms that organisms in mangrove soils use to adapt to their unique challenges in the environment.

### Gene enrichment analysis

**Fig. 7:**
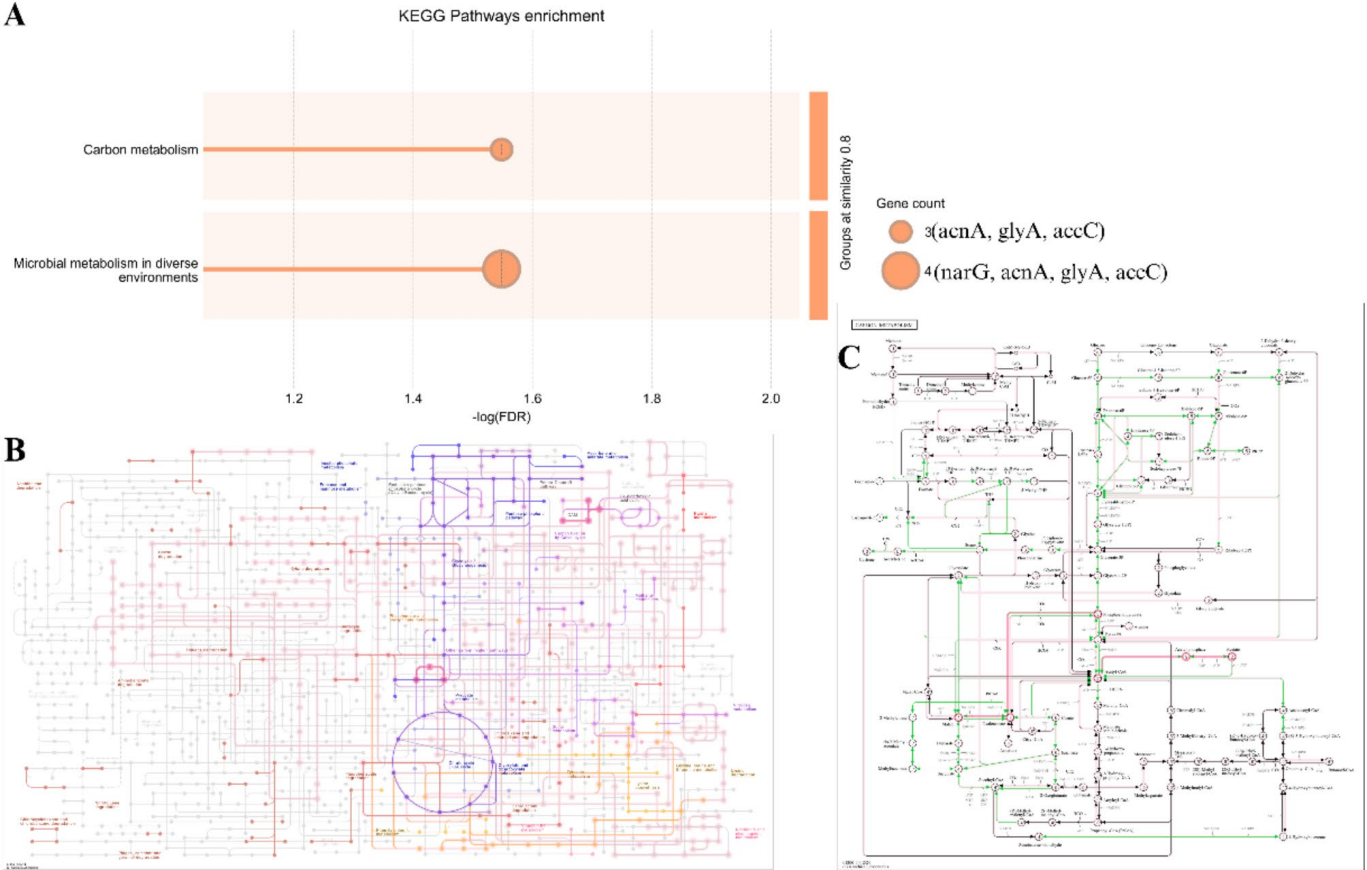
Diagram of the carbon cycle path derived from annotated carbon cycle genes. (**A**) KEGG pathway enrichment, (**B**) Carbon fixation pathway, (**C**) Carbon metabolism pathway.

The KEGG pathway enrichment analysis identified two distinct groups associated with carbon regulation: carbon metabolism and microbial metabolism in different environments. The carbon metabolism group exhibited a significant enrichment in the gene counts of acnA, glyA, and accC, underscoring their pivotal function in the control and processing of carbon intermediates. The microbial metabolism in multiple environments group exhibited an enrichment of gene counts for narG, acnA, glyA, and accC, indicating their role in carbon control across different environmental circumstances, as seen in Fig. 7A. Furthermore, the carbon fixation process is highlighted in Fig. 7B, focusing on two main modules: Crassulacean Acid Metabolism (CAM) and the Phosphate Acetyltransferase-Acetate Kinase (PTA-AK) pathway. These pathways are essential for carbon fixation in ecosystems that use alternate carbon sources. Figure 7C depicts the whole carbon metabolism path, provides an overview of the interrelated processes of carbon regulation.

## Discussion

The metagenomic study of soil samples from both mangrove and non-mangrove areas provided valuable information on the variety of microorganisms and their probable functions in these ecosystems. Other research has also shown the significance of microbial communities in different environmental contexts, highlighting the functions of certain genes and metabolic pathways in the functioning of ecosystems. The investigation revealed clear differences in the microbial populations found in mangrove and non-mangrove soils, consistent with previous research^25^, which also found significant differences in microbial diversity in mangrove soils. However, our study extends this finding by identifying specific CRGs associated with carbon cycling, such as RuBisCO and urease, which were less emphasized in earlier studies. Our findings align with those of, who reported similar patterns of gene diversity in coastal ecosystems, but we also observe unique functional pathways in mangrove soils, potentially due to the influence of salinity and tidal flooding^26^. Bacteria, proteobacteria, and viruses were found in different quantities in each type of soil. These results align with the findings, who observed a significant level of microbial diversity in mangrove soils^27^. This emphasizes the impact of environmental conditions on the organization of microbial communities. The identification of various CRGS, such as Tetrahydrofolate ligase, Carboxylase, Ribulose-1,5-bisphosphate carboxylase/oxygenase, Glycine hydroxymethyltransferase, MAG: urease, Endosymbiont of Oligobrachia haakonmosbiensis, Ribulose bisphosphate carboxylase, Aconitate hydratase AcnA, Triacylglycerol lipase, NarG, DsrB, DNA-binding transcriptional dual regulator CRP, Vanillate O-demethylase oxygenase, succinate-CoA ligase, and nitrous oxide reductase, highlights the mangrove soils may exhibit gene distributions suggesting potential adaptations to salinity and tidal influence. Research was shown that mangrove soils serve as focal points for microbial activity, namely in the process of carbon cycling^2,28^. This activity plays a crucial role in preserving the health and resilience of ecosystems. We suggest utilizing transcriptomics and proteomics to confirm the expression and functional roles of these genes in the microbial communities of mangrove soils.

Metagenomic techniques have played a crucial role in revealing the intricate microbial communities present in different settings. For example, metagenomics to investigate soil microbial communities and their functional capabilities^29,30^. Their findings demonstrated comparable patterns in gene diversity and metabolic functions to those seen in our work. The assembly statistics of soil samples from mangrove and non-mangrove areas provide a thorough assessment of the quality and intricacy of the genetic data. The scaffold and contig metrics observed are comparable to those described in a study of microbial communities in different soil types, reinforcing the robustness of our assembly and quality control processes^31^. Research has shown that mangrove ecosystems play a crucial role in capturing and storing CO_2_ because of their exceptional ability to produce organic matter and effectively store carbon in the sediment. Our results are consistent with the research, which highlighted the significance of microbial activity in the carbon cycling of mangroves^2^. The identification of genes associated with carbon degradation and transformation, including those involved in carbon fixation (e.g., RuBisCO) and organic matter decomposition (e.g., triacylglycerol lipase), shows the direct impact of microbial communities on carbon cycling^32^. Genes associated with carbon fixation boost CO_2_ sequestration in mangrove soils, hence aiding in the reduction of atmospheric carbon levels. Conversely, genes associated with organic matter breakdown facilitate the release of sequestered carbon into the soil, hence enhancing nutrient cycling. These microbial mechanisms are crucial for sustaining ecosystem stability, since they regulate the carbon exchange among the atmosphere, soil, and biomass. Comprehending these microbial roles helps the development of more informed management methods aimed at improving carbon sequestration and ecosystem health, particularly in carbon-rich ecosystems such as mangroves^33^. The discovered core functional gene clusters CRGs showcase the wide range of mangrove soils may exhibit gene distributions suggesting potential adaptations to salinity and tidal influence^34^.

The study includes bacteria or enzymes that cover a wide range of biochemical pathways and activities, showcasing the variety of enzymatic activity in microbial metabolism and carbon management. Comprehending these enzymes is essential for comprehending the metabolic networks and ecological roles of microorganisms, particularly in symbiotic relationships or specialized environments. Tetrahydrofolate Bacterial ligase plays a crucial role in folate metabolism by facilitating the activation of tetrahydrofolate (THF) by ligation with ATP. Tetrahydrofolate (THF) plays a critical role in facilitating one-carbon transfer events, which are vital for nucleotide synthesis and amino acid metabolism^35^. Carboxylase is a group of enzymes found in plants that facilitate the addition of CO_2_ to various substances. An exemplary instance is Ribulose-1,5-bisphosphate carboxylase/oxygenase (RuBisCO), which enables the first stage of carbon fixation in the Calvin cycle of photosynthesis. The activity of RuBisCO is essential for the conversion of inorganic carbon into organic forms, which is a critical mechanism for regulating carbon in photosynthetic organisms^36^. Glycine is an amino acid. Hydroxymethyltransferase, or serine hydroxymethyltransferase, is crucial in the transformation of serine and glycine, aiding in the folate cycle and the production of nucleotides. The enzyme’s function indirectly contributes to the control of carbon by participating in the metabolism of amino acids and the conversion of carbon units^37^. Urease, also known as MAG, is an enzyme that facilitates the breakdown of urea into CO_2_ and ammonia. This process is crucial for nitrogen metabolism in many species, including plant symbionts such as rhizobia. The generation of CO_2_ as a secondary result connects urease action to the wider carbon cycle^38,39^. Nitrous Oxide Reductase is the last stage in the denitrification process, when it converts nitrous oxide (N_2_O) into nitrogen gas (N_2_). The reduction process indirectly impacts carbon control by influencing the balance of greenhouse gases in the atmosphere, while its principal purpose is in nitrogen management^40^. The endosymbiont of Oligobrachia haakonmosbiensis bacteria was derived from the endosymbiotic bacteria found in the sea worm Oligobrachia haakonmosbiensis. The symbionts enhance the host’s metabolic capacity, especially in conditions with limited nutrients, by supplying enzymes that are involved in carbon fixation and the digestion of organic carbon^38,40,41^. Aconitase, also known as aconitate hydratase (AcnA), is an enzyme found in the citric acid cycle. Its main function is to facilitate the conversion of citrate into isocitrate by isomerization. It has a vital function in the creation of cellular energy and control of carbon by aiding in the conversion of carbon substrates through the cycle^42^. The Triacylglycerol Lipase bacterium enzymatically breaks down triacylglycerols into glycerol and free fatty acids, playing a crucial role in cellular lipid metabolism and energy mobilization. Lipid breakdown liberates carbon atoms that may be used in several metabolic pathways, hence contributing to carbon control^42^. NarG served as a constituent of the nitrate reductase complex, which played a role in converting nitrate to nitrite throughout the nitrogen cycle. DsrB was a component of the dissimilatory sulfite reductase complex, contributing to sulfur metabolism. Both enzymes have a role in the nitrogen and sulfur cycles, which are associated with carbon cycling in the environment. As a result, they indirectly impact carbon control^40,41^. The Vanillate O-demethylase Oxygenase enzyme played a role in the decomposition of substances generated from lignin, especially by facilitating the demethylation of vanillate. This demethylation phase is part of the process of breaking down aromatic compounds. This technique facilitates the liberation of carbon atoms from intricate organic compounds, hence contributing to the management and turnover of carbon in the environment^36,37^. Succinate–CoA Ligase, an enzyme involved in the citric acid cycle, facilitates the conversion of succinate to succinyl-CoA, hence contributing to the generation of ATP and the regulation of intermediate metabolism. It directly contributes to the control of cellular carbon by aiding in the conversion of carbon substrates^38,39^.

The enrichment of acnA, glyA, and accC in carbon metabolic pathways aligns with the results of Kurt et al. (2018)^43^, who illustrated their function in enhancing carbon flux via the TCA cycle and one-carbon metabolism. The participation of narG and acnA in microbial metabolism across various habitats corroborates the findings of Green & Guest, (1998)^44^, emphasizing their resilience to fluctuating ecological situations. The emphasized Crassulacean Acid Metabolism (CAM) and PTA-AK pathways align with the findings of Dodd et al., (2002)^45^, Liu et al., (2006)^46^, underscoring their significance in alternate carbon fixation mechanisms in resource-constrained environments.

The findings of Shyam et al. (2023) align with our results, since they showed how soil moisture affects the composition of microbial communities and the presence of carbon cycling genes in various soil types^47,48^. The existence of comparable functional genes in many soil conditions highlights the widespread involvement of microorganisms in controlling carbon cycles. The metagenomic profiles of soils from mangrove and non-mangrove areas provide detailed insights into the complex functions of microbial communities in carbon management. Through a comparative analysis of our results with earlier research, we emphasize the significant role that microorganisms play in the processes of carbon cycling. This highlights the need for more investigations to examine these dynamics in other environmental situations.

## Materials and methods

### Sampling sites and sediment collection

Sediment samples were taken from mangrove regions in Jharkhali, situated in the Basanti block of South 24 Parganas, West Bengal, India, inside tidal plantation zones between coordinates 22° 01′ 09″ N and 88° 40′ 56″ E, with a salinity of 9 g/L. Soil samples of non-mangrove, were collected from a harvested paddy field in Sonatikari, South 24 Parganas, West Bengal, India, located at coordinates 22° 01′ 24.0″ N and 88° 30′ 19.1″ E, with a salinity of 1 g/L. Sampling involved five spots within each site, strategically selected to capture spatial heterogeneity. Approximately 30 g of soil from each spot was collected using a Van Veen grab (from the top 20 cm of the benthic layer, focusing on the soil). Soil from the five spots within each site was pooled to create a single composite sample, ensuring a comprehensive representation of microbial diversity and reducing site-specific variability^5^. Each location produced one soil grab, and grain size was evaluated using particle sieve analysis. Two sediment samples from each environment were combined for analysis. Descriptive graphical visualization was shown in Fig. 1.

### Extraction of genomic DNA

DNA was isolated from the pooled soil samples using the conventional phenol–chloroform technique. On average 200 ng of the extracted DNA was subjected to analysis on a 0.8% agarose gel, performed at 120 V for 60 min till the sample traversed three-quarters of the gel’s length. To analyze the quality and concentration of the DNA, 1 µl of each sample was assessed using a Nanodrop 2000 spectrophotometer to ascertain the A260/280 ratio, while a Qubit 3.0 Fluorometer was used to quantify the genomic DNA. Subsequently, high-quality genomic DNA was used to generate next-generation sequencing libraries.

### Library preparation and quality control

The illuminaTruSeq Nano DNA Library Preparation kit was used to generate paired-end sequencing libraries from QC-passed. The fragments have gone through end-repair techniques. This approach results in a low rate of chimera (concatenated template) creation. The ligated products were size-selected using AMPure XP beads. These size-selected components went through PCR amplification. The PCR-enriched library was analyzed using the 4200 Tape Station System (Agilent Technologies) with high sensitivity D1000 Screen tape, following the manufacturer’s recommended protocols.

### Cluster generation and sequencing

The Pair-End illumina library was loaded into NextSeq 500 for cluster generation and sequencing, using Agilent Tape Station profile for Qubit concentration and peak size. The library fragments were sequenced forward and reverse, with mean fragment size distributions for mangrove and non-mangrove groups at 448 bp and 419 bp, respectively^5^. The highly curated raw reads of soil samples were deposited in NCBI-SRA database using BioProject IDs PRJNA436903 (https://www.ncbi.nlm.nih.gov/sra/?term=SRR6811659) and PRJNA437568 (https://www.ncbi.nlm.nih.gov/sra/?term=PRJNA437568).

### Trimming, and Denovo assembly of metagenome data

In order to eliminate adapter sequences, ambiguous reads (reads with unknown nucleotides “N” larger than 5%), and low-quality sequences (reads with more than ten quality thresholds (QV) < 20 phred score), the sequenced raw data was processed using fastp v 0.23.2 to obtain high-quality clean reads. A minimum length of 100 nts was permitted following the procedure of trimming. 40,877,196 (2 × 150 bp) and 34.413,814 (2 × 150 bp) high-quality reads were obtained for mangrove and non-mangrove samples, respectively, after the adapter and low-quality sequences were removed from the raw data. MEGAHIT v 1.1.3 was employed to construct scaffolds from the filtered high-quality reads of the mangrove and non-mangrove samples, with the following parameters minimum multiplicity for filtering (k_min + 1)-mers = 2 and minimum length of contig was set as 200 and other parameters was set as default.

### Analysis of microbial annotation and taxonomic classification

The predicted genes from both samples were taxonomically classified using the Kraken 2 (v 2.1.3) tool. The background Kraken database was “standard plusPF v: 2024-01-12” and the other parameters was set as default. This program is designed for accurate classification of high throughput metagenomic sequencing data from the NCBI Refseq or the microbial subset of the NCBI BLAST non-redundant protein database. It can also include fungi and microbial eukaryotes.

### Carbon regulating microbial gene identification

The dataset of carbon regulating genes (CRGs) was compiled by conducting a thorough literature review of both mangrove (8995) and non-mangrove (13792) species. The data underwent a thorough process of rechecking and verification using the NCBI searches. We used the curated dataset of genes involved in carbon regulation as the baseline dataset for our analysis aimed at identifying carbon regulating genes (CRGs). We have used a conventional tBLASTn with an expected cut-off value of 0.001 to identify microbial carbon regulating genes in our metagenome. The BLAST result underwent analysis and filtration using several metrics including percent identity, query coverage, and BLAST score.

### Characterization of genes involved in carbon regulation using functional profiling

The Kyoto Encyclopedia of Genes and Genomes (KEGG) database was employed to annotate functional genes and metabolic pathways in the metagenomic dataset. Functional annotation was performed by mapping sequences to the closest matching reference sequences, based on the best-hit approach using MEGAN V6^48^. The Clusters of Orthologous Groups (COG) database classifies proteins into functional categories according to orthology, facilitating the functional annotation of genes in metagenomic research. The SEED database offers functional annotations and subsystem classifications, facilitating the identification of metabolic pathways and functional capabilities within metagenomic datasets. Functional analysis was performed with MEGAN V6^48^. The parameters were adjusted in default. The Microsoft PowerBI program was used for plot visualization, allowing for the concurrent annotation of individual sequences with KEGG, COG, and SEED subsystem annotations.

### Enrichment analysis

All the identified carbon regulating gene was used for enrichment analysis using string () with background database; *Escherichia coli*. KEGG pathway enrichment plot was grouped by similarity value ≥ 0.08 and sort with -log10 (fdr) value. Ethe enriched pathway diagram was retrieved from KEGG pathway () database.

### Analysis of statistical data

In order to examine the data about genes that regulate carbon in the Sundarban mangrove (MG) and non-mangrove (NMG) groups, we performed an independent samples significant t-test using the R statistical software package. We computed the descriptive statistics for each group, which included the mean, standard deviation (SD), standard error (SE), and margin of error (ME). The t-test indicated a mean difference of 1.333 across the groups, with a p-value of 0.649.

### Limitations and perspectives

The study aimed to explore soil microbial diversity and functional potential at two separate locations through a pooled sampling method. Pooling samples is a prevalent technique for capturing regional variation and representing the entire microbial environment. Despite these limitations, the pooling methodology used in this work enabled the integration of microbiological signals from five meticulously chosen locations within each site, therefore providing a representative overview of the sediment environment. This technique, while cost-effective, provides critical baseline data on microbial diversity and function in understudied ecosystems. Subsequent research may expand upon these results by integrating biological replicates and longitudinal sampling to enhance comprehension of temporal and spatial variability.

## Conclusion

The thorough metagenomic investigation of soils in the Sundarban mangrove and non-mangrove areas has shown the unique microbial populations and their functional capabilities in these ecosystems. The findings emphasize the intricate and varied nature of microbial life in various habitats, emphasizing notable variations in microbial makeup, with a greater proportion of GC content and distinct taxonomic groups such as Actinobacteria and Firmicutes being prominent in mangrove soils. The quality control and assembly statistics provide as evidence of the durability of our sequencing and analytical techniques, ensuring dependable data for further analysis. The identification of various carbon-regulating genes (CRGs), including well-known genes like Tetrahydrofolate ligase and Ribulose-1,5-bisphosphate carboxylase/oxygenase, as well as genes unique to mangrove and non-mangrove soils, suggests potential differences in microbial metabolic capabilities. However, due to the lack of statistically significant differences in CRG abundance (*p* > 0.05), these findings should be considered as observed trends rather than definitive ecological insights. Further statistical analysis and experimental validation are needed to better understand the ecological significance of these microbial processes in the context of mangrove and non-mangrove ecosystems. CRGs, or carbon-related genes, have crucial functions in the carbon cycle, playing important roles in activities like as carbon fixation and the breakdown of organic matter. These processes are vital for maintaining the health and resilience of ecosystems. The taxonomic profile and functional analysis corroborate prior research, emphasizing the crucial role of microbial communities in carbon management across various soil conditions. The existence of particular metabolic pathways and the observed microbial diversity highlight the substantial role that soil microorganisms play in carbon sequestration and the general functioning of ecosystems. Further investigation is needed to explore the specific environmental elements that impact the architecture and functional capabilities of microbial communities. This research will improve our understanding of the role microorganisms play in the carbon cycle and the ability of ecosystems to adapt to changing environmental circumstances. The knowledge acquired from this work establishes a basis for future research on the ecological functions of microorganisms in both mangrove and non-mangrove soils. This will facilitate the development of improved methods for managing and conserving these crucial ecosystems.

## Electronic supplementary material

Below is the link to the electronic supplementary material.

Supplementary Material 1

## Data Availability

The highly curated raw reads of soil samples were deposited in NCBI-SRA database using BioProject IDs PRJNA436903 (https://www.ncbi.nlm.nih.gov/sra/?term=SRR6811659) and PRJNA437568 (https://www.ncbi.nlm.nih.gov/sra/?term=PRJNA437568).
