# Supplementary material for: Exploring microbial players for metagenomic profiling of carbon cycling bacteria in sundarban mangrove soils
Source: Sci Rep. 2025 Feb 8;15:4784. doi: 10.1038/s41598-025-89418-x (PMC11807184; doi:10.1038/s41598-025-89418-x)
Supplement: Supplementary file 1 — Supplementary Material 1 [file 41598_2025_89418_MOESM1_ESM.docx]

**Table S1:** Quality Control of Sundarban mangrove and non-mangrove

| Category | Metric | SRR6811659 (Mangrove) | SRR6821719 (Non-Mangrove) |
| --- | --- | --- | --- |
| General | fastp version | 0.23.4 (https://github.com/OpenGene/fastp) | 0.23.4 (https://github.com/OpenGene/fastp) |
|  | Sequencing | paired end (151 cycles + 151 cycles) | paired end (151 cycles + 151 cycles) |
|  | Mean length before filtering | 148bp (read1), 147bp (read2) | 147bp (read1), 147bp (read2) |
|  | Mean length after filtering | 148bp (read1), 147bp (read2) | 148bp (read1), 147bp (read2) |
|  | Duplication rate | 0.54% | 0.74% |
|  | Insert size peak | 216 | 204 |
| Before filtering | Total reads | 81.754392 M | 68.827628 M |
|  | Total bases | 12.094831 G | 10.169058 G |
|  | Q20 bases | 10.580338 G (87.478186%) | 9.071920 G (89.211014%) |
|  | Q30 bases | 9.283210 G (76.753536%) | 8.041015 G (79.073351%) |
|  | GC content | 51.54% | 63.96% |
| After filtering | Total reads | 80.422410 M | 67.855854 M |
|  | Total bases | 11.911333 G | 10.033421 G |
|  | Q20 bases | 10.462141 G (87.833506%) | 8.981301 G (89.513845%) |
|  | Q30 bases | 9.187546 G (77.132815%) | 7.966785 G (79.402479%) |
|  | GC content | 51.54% | 63.96% |
| Filtering result | Reads passed filters | 80.422410 M (98.370752%) | 67.855854 M (98.588105%) |
|  | Reads with low quality | 1.321976 M (1.617009%) | 961.268000 K (1.396631%) |
|  | Reads with too many N | 10.006000 K (0.012239%) | 10.506000 K (0.015264%) |
|  | Reads too short | 0 (0.000000%) | 0 (0.000000%) |
|  | | |  |

**Table S2:** Assembly statistics for mangrove and non-mangrove soil sample

| Assembly features | Mangrove | Non-mangrove |
| --- | --- | --- |
| Scaffold L50 | 257940 | 291040 |
| Scaffold N50 | 627 | 622 |
| Scaffold L90 | 886016 | 924552 |
| Scaffold N90 | 343 | 348 |
| Scaffold len_max | 271595 | 153356 |
| Scaffold len_min | 200 | 200 |
| Scaffold len_mean | 635 | 627 |
| Scaffold len_median | 441 | 456 |
| Scaffold len_std | 1287 | 979 |
| Scaffold num_A | 1.78E+08 | 1.3E+08 |
| Scaffold num_T | 1.73E+08 | 1.28E+08 |
| Scaffold num_C | 1.76E+08 | 2.32E+08 |
| Scaffold num_G | 1.76E+08 | 2.31E+08 |
| Scaffold num_N | 0 | 0 |
| Scaffold num_bp | 7.03E+08 | 7.2E+08 |
| Scaffold num_bp_not_N | 7.03E+08 | 7.2E+08 |
| Scaffold num_seq | 1106628 | 1148660 |
| Scaffold GC content overall | 50.1 | 64.24 |
| Contig L50 | 257940 | 291040 |
| Contig N50 | 627 | 622 |
| Contig L90 | 886016 | 924552 |
| Contig N90 | 343 | 348 |
| Contig len_max | 271595 | 153356 |
| Contig len_min | 200 | 200 |
| Contig len_mean | 635 | 627 |
| Contig len_median | 441 | 456 |
| Contig len_std | 1287 | 979 |
| Contig num_bp | 7.03E+08 | 7.2E+08 |

**Table S3** A t-test to compare the means of carbon regulating genes between two groups: Sundarban mangrove (MG) and non-mangrove (NMG).

| Group | Mean | number | SD | SE | ME | Difference (NMG - MG) | p-value |
| --- | --- | --- | --- | --- | --- | --- | --- |
| NMG | 6.333 | 15 | 10.161 | 2.623 | 5.627 | 1.333 | 0.649 |
| MG | 5 | 15 | 4.645 | 1.199 | 2.572 |  |  |
